# Controllable Cu^+^/Cu^2+^ ratio for the gas-sensing property of (Na, Cu) co-doped ZnO investigated by EPR and SPV

**DOI:** 10.1039/d5ra04390e

**Published:** 2026-02-16

**Authors:** Qiong Zhang, Liyao Wen, Yifei Sun, Yunkuan Zhao, Huan Yuan, Yaxi Chen, Fei Yu, Kang Zhao, Ming Xu

**Affiliations:** a Key Laboratory of Information Materials of Sichuan Province, School of Electronic Information, Southwest Minzu University Chengdu 610041 China yuanhuanwill@126.com yufei@swun.edu.cn; b National Laboratory of Solid State Microstructures, Nanjing University Nanjing 210093 China; c School of Computer and Artificial Intelligence, Southwest Minzu University Chengdu 610041 China yaxichen@swun.cn

## Abstract

Herein, (Na, Cu) co-doped ZnO nanocrystals were prepared using the sol–gel method. By carefully balancing the Cu^+^/Cu^2+^ ratio through Na doping in Zn_0.95_Cu_0.05_O-based sensors, the gas-sensing activity can be significantly enhanced. Interestingly, we found that increasing Na doping results in the transformation of Cu^2+^ to Cu^+^ in the copper ion valence state. Furthermore, the XPS results indicate that Na^+^ ions increase the oxygen vacancies of the samples, which is in agreement with the electron paramagnetic resonance (EPR) results. The surface photovoltage (SPV) spectra indicate that (Na, Cu) co-doped ZnO nanocrystals with a high Cu^+^/Cu^2+^ ratio exhibit a high positive SPV response, demonstrating the excellent separation efficiency of photogenerated charges. Oxygen vacancies and the transformation of Cu^2+^ to Cu^+^ are presumed to be the driving factors responsible for UV light-activated NO_2_ gas-sensing improvement in this study. This work demonstrates a strategy for improving the gas-sensing efficiency of ZnO-based sensors through leveraging the variable valence states of incorporated metal species.

## Abstract 2

Increased Na doping promotes the transformation of Cu^2+^ to Cu^+^ and introduces more oxygen vacancies of ZnO-based sensors through (Na, Cu) co-doping for room-temperature NO_2_ detection with a limit as low as 250 ppb.

## Graphical abstract

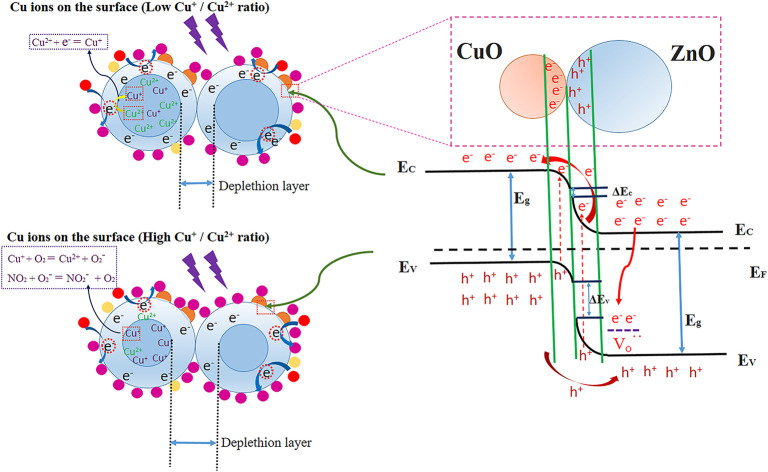

## 1. Introduction

Zinc oxide (ZnO) is a significant n-type semiconductor material with a wide band gap of 3.37 eV,^1,2^ which is widely used in the development of diode devices,^3–5^ photodetectors,^6^ and antibacterial agents.^7,8^ Inorganic metal oxides like TiO_2_,^9,10^ MgO,^11^ ZnO,^12–14^ and CuO^15^ exhibit biocompatibility as well as excellent chemical and thermal stability. Doping ZnO with transition metals such as Mg, Co, Cu, and Ni has been shown to enhance its physical, chemical, and antibacterial properties, thereby expanding its potential for various applications.^16,17^ Using Cu-doped polycrystalline ZnO deposited on glass substrates, it has been found that refractive indices increase and forbidden energy gap values decrease with increasing Cu dopant concentration. The enhanced degradation performance of Cu-doped ZnO synthesized with a facile solution route can be attributed to hierarchical nanostructures and the formation of an acceptor level (Cu^2+^–Cu^+^), compared with pristine ZnO.^19^ According to the abovementioned literature, the valence state of Cu has a strong influence on the microstructure performance of the samples. Brahma^20^ synthesized Cu-doped ZnO nanowires *via* chemical vapor deposition and demonstrated striking responses to acetone gas at 1 ppm at room temperature for only the p-type Cu-doped ZnO. Interestingly, the variation of n-type Cu-doped ZnO to p-type is due to the successful substitution of Cu^+^ for Zn^2+^ lattice sites *via* Cu doping. Ganesh systematically studied the Cu transition metal for enhancing the response and recovery time of the gas sensor, and Cu-doped ZnO (6 wt%) nanoflowers showed enhanced selectivity toward ammonia at a 10-ppm concentration.^21^ One unresolved issue is that the effects of tuning and probing Cu valence states remain insufficiently explored. Fabrication of Na-doped ZnO nanowires, as potential materials of blue emission, *via* a thermal decomposition route led to a strong photoluminescence band with a major peak at 420 nm.^22^ Na as a substitutional group-I element is a shallow acceptor for the Na-doped ZnO, which is able to inhibit enzymes and facilitate the generation of reactive oxygen species. In Sáaedi's study,^23^ undoped and Na-doped ZnO nanorods were prepared, and their gas-sensing performance against ethanol was evaluated. Compared with pure ZnO, surface oxygen vacancies induced by Na doping are presumed to be a driving factor behind the improvement of gas-sensing efficiency in Na-doped ZnO nanorods. Meanwhile, Na-doped ZnO prepared using a simple precipitation method exhibited a highly efficient photocatalytic performance for the methylene blue dye compared with pure ZnO and is considered a promising photocatalyst based on density functional theory calculations.^24^ Jaisutti^25^ argued that p-type Na-doped ZnO nanoflowers with hierarchical nanosheets were synthesized as acetone gas sensors with a fast response time of 18 s (100 ppm), which enables the diagnosis of various diseases, including diabetes, from exhaled breath. In this regard, Na-doped ZnO is worthy of further study to illuminate the behavior of sodium in ZnO.

To date, many studies on Cu- or Na-doped ZnO have focused on the influence of Cu or Na doping alone on photocatalytic activities or on the nanostructure and electrical properties at different doping concentrations. As far as we know, there are virtually no reports on the interfacial effects of Na ion doping on the gas-sensing performance of the Zn_0.95−*x*_Cu_0.05_Na_*x*_O nanocrystalline. In our study, we propose whether it is possible to further enhance gas-sensing performance by tuning the Cu^+^/Cu^2+^ ratio as a facile way to enrich the surface active sites of (Na, Cu) co-doped ZnO. Specifically, by precisely controlling the doping concentration of Na^+^ ions to modulate the electron transfer between Cu^+^ and Cu^2+^, and by leveraging the synergistic redox effects arising from heterostructure formation and surface deep-level defects, the photo-excited carrier lifetime is significantly prolonged, leading to enhanced NO₂ gas-sensing performance.^18^

## 2. Experimental

### 2.1 Synthesis of Zn_0.95−*x*_Cu_0.05_Na_*x*_ nanocrystals

Na-doped Zn_0.95_Cu_0.05_O nanocrystals were prepared using the sol–gel method. Sodium acetate trihydrate, zinc nitrate hexahydrate, and copper acetate monohydrate were the individual sources of Na, Zn and Cu, respectively. The above chemicals were added to a suitable alcohol in a beaker to form a homogeneous precursor solution, which was incubated in a water bath at 60 °C for 2 hours under magnetic stirring. A stabilizer (2-ml ethanolamine) was added to the precursor, and the pH of the precursor was adjusted to 8 using glacial acetic acid or ammonia. The solution was incubated for 72 hours under dark conditions at room temperature. The resultant gel was dried at 80 °C for 8 hours in a dryness box and then calcined in a muffle furnace at 800 °C for 6 hours in an air atmosphere. The fabricated samples were denoted as Zn_0.95−*x*_Cu_0.05_Na_*x*_O nanocrystals (*x* = 0, 0.005, 0.01, 0.02, 0.05, and 0.1), corresponding to the molar ratios of CH_3_COONa to Zn(NO_3_)_2_.

### 2.2 Sample characterization

An X-ray automatic diffractometer (DX-2000) was used to characterize the nanocrystals' phase compositions with a step size of 0.02°. The surface morphology of the samples was investigated using a transmission electron microscope (JEOL JEM2100PLUS) operating at an acceleration voltage of 200 kV and a scanning electron microscope (HITACHI Regilus8100). The surface chemical composition was identified using an X-ray photoelectron spectrometer (VG ESCALAB 210) calibrated to the C 1s peak of 285.0 eV for the binding energies. The spectrum decomposition was analyzed using the Gaussian functions of the XPS PEAK program. N_2_ adsorption and desorption isotherms were measured using the Micromeritics ASAP2460 Surface Area and Porosimetry Analyzer, where all polymer samples were degassed at 110 °C for 10 h under a vacuum of 10^−5^ bar prior to analysis. The BET surface area was evaluated based on the N_2_ adsorption and desorption isotherms using the BET equation. Electron paramagnetic resonance (EPR) measurements were made using a Germany E500 spectrometer to further characterize the intrinsic defects of Zn_0.95−*x*_Cu_0.05_Na_*x*_O nanocrystals at room temperature. The surface photovoltage (SPV) spectra were measured using a custom-made instrument containing a lock-in amplifier (SR830-DSP), a light chopper (SR540) and a photovoltaic cell in our laboratory.

### 2.3 Gas-sensing measurements

**Fig. 1:**
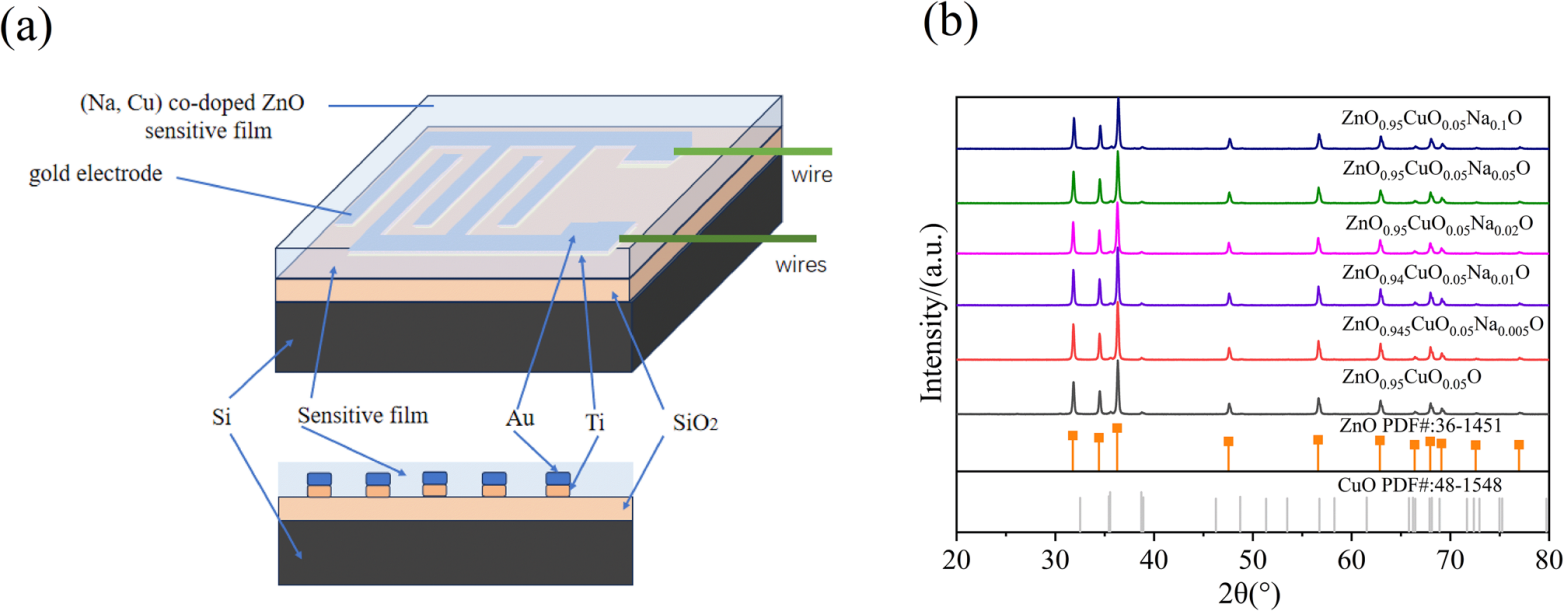
(a) Structure and cross-section of the as-prepared sensors; (b) XRD pattern of the Zn_0.95−*x*_Cu_0.05_Na_*x*_O nanocrystals (*x* = 0, 0.005, 0.01, 0.02, 0.05, and 0.1).

Au interdigitated electrodes on an Si substrate were fabricated using a micro-electro-mechanical system (MEMS) technology.^26^Fig. 1(a) presents the structure and cross-section of the as-prepared sensors. An interdigitated electrode was fabricated through the following steps: first, a 500-nm-thick silicon dioxide was thermally grown on Si (100) as AN insulating layer. Second, a 200-nm-thick titanium and a 500-nm-thick gold were deposited *via* thermal evaporation. Third, the IDE with a finger width of 50 µm and a gap width of 50 µm was prepared *via* conventional photolithography and the lift-off method. For cleaning the Si substrate, acetone, ethanol, and deionized water were added in turn, and nitrogen gas stream was used to dry the substrate. Ethanol suspensions containing fabricated nanocrystals (3.0 mg ml^−1^) were added to the Si substrate *via* spin coating. UV LED light sources (365 nm, Taiwan Light Macro Chip) and the Keithley 2700 data acquisition system were used for gas-sensing measurement. The sensor response was calculated as (*R*_g_ – *R*_0_)/*R*_0_, where *R*_g_ is the real-time resistance exposure to NO_2_ and *R*_0_ is the real-time resistance of dry air from a compressed cylinder.^27^ Sensitivity was defined as the slope of the response-concentration fitting curve.^28^

## 3. Results and discussion

### 3.1 Material structural studies

To uncover the physical basis for the gas sensing and antibacterial properties of the nanocrystals, the microstructure of Zn_0.95−*x*_Cu_0.05_Na_*x*_O nanocrystals was characterized *via* X-ray diffraction (XRD). Two sets of diffraction peaks are shown in Fig. 1(b). The strong and sharp diffraction peaks of hexagonal wurtzite ZnO are visible in the XRD pattern, providing evidence that the fabricated samples are well-crystallized. Previously, we reported (Co, Cu)-co-doped ZnO and did not observe CuO peak,^29^ which could be due to the high-temperature treatment and long treatment time. This work presents a strategy for synthesizing oxide composite nanomaterials. Meanwhile, there were slight shifts in the peak positions of the Zn_0.95−*x*_Cu_0.05_Na_*x*_O nanocrystals with the introduction of Na. The Na ions prefer to aggregate at interstitial sites because the ionic radius of Na^+^ (1.02 Å) is larger than that of Zn^2+^ (0.74 Å).^30^ Cu^2+^ (0.73 Å) tends to occupy Zn ion sites because of the similar radii of Cu and Zn. Therefore, these data confirm that Na and Cu are incorporated into the Zn–O lattice.

**Fig. 2:**
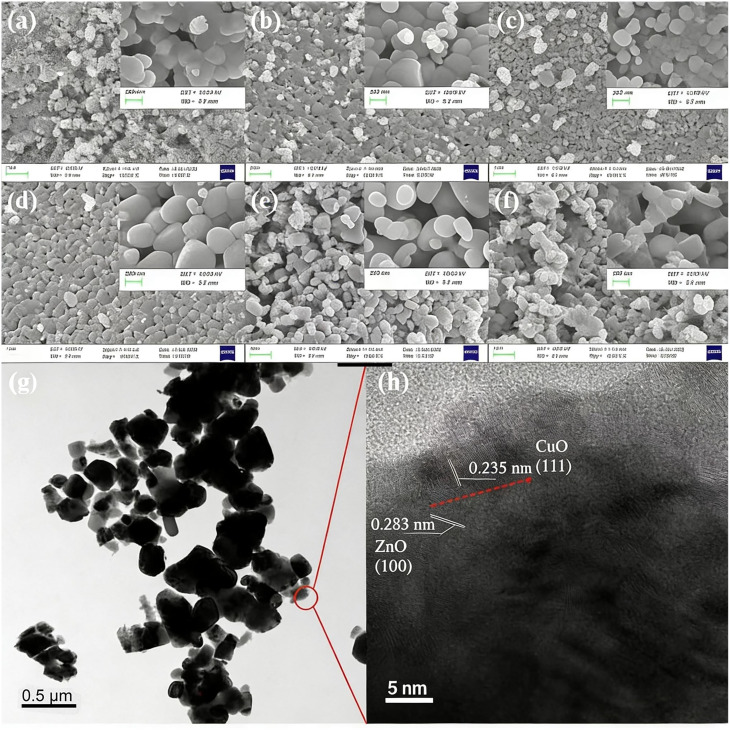
SEM images of the Zn_0.95−*x*_Cu_0.05_Na_*x*_O nanocrystals prepared at (a) *x* = 0, (b) *x* = 0.005, (c) *x* = 0.01, (d) *x* = 0.02, (e) *x* = 0.05, (f) *x* = 0.1; and (g and h) TEM images of the Zn_0.9_Cu_0.05_Na_0.05_O nanocrystals with different enlargements.

**Fig. 3:**
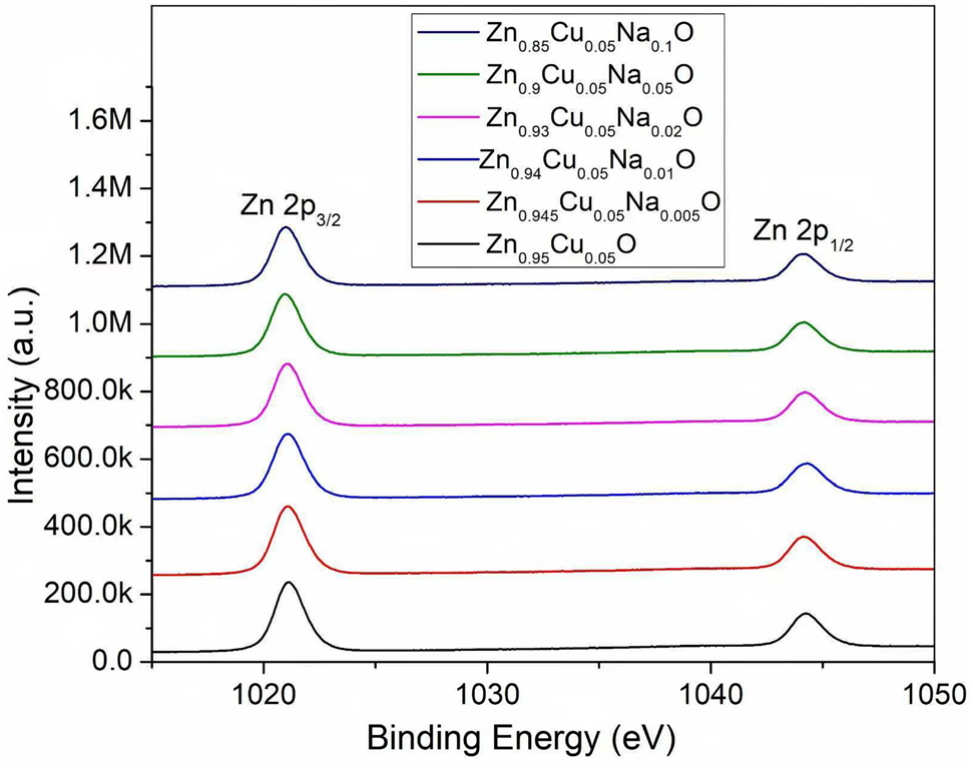
XPS of the Zn 2p core level of the Zn_0.95−*x*_Cu_0.05_Na_*x*_O nanocrystals.

**Fig. 4:**
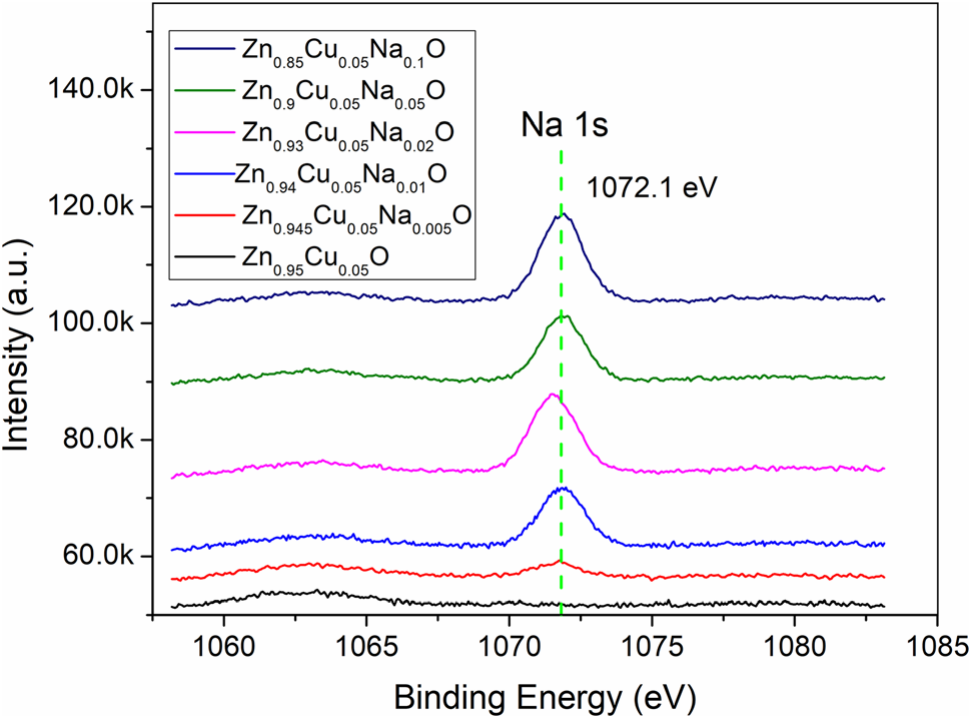
XPS of the Na 1s core level for the Zn_0.95−*x*_Cu_0.05_Na_*x*_O nanocrystals.

**Fig. 5:**
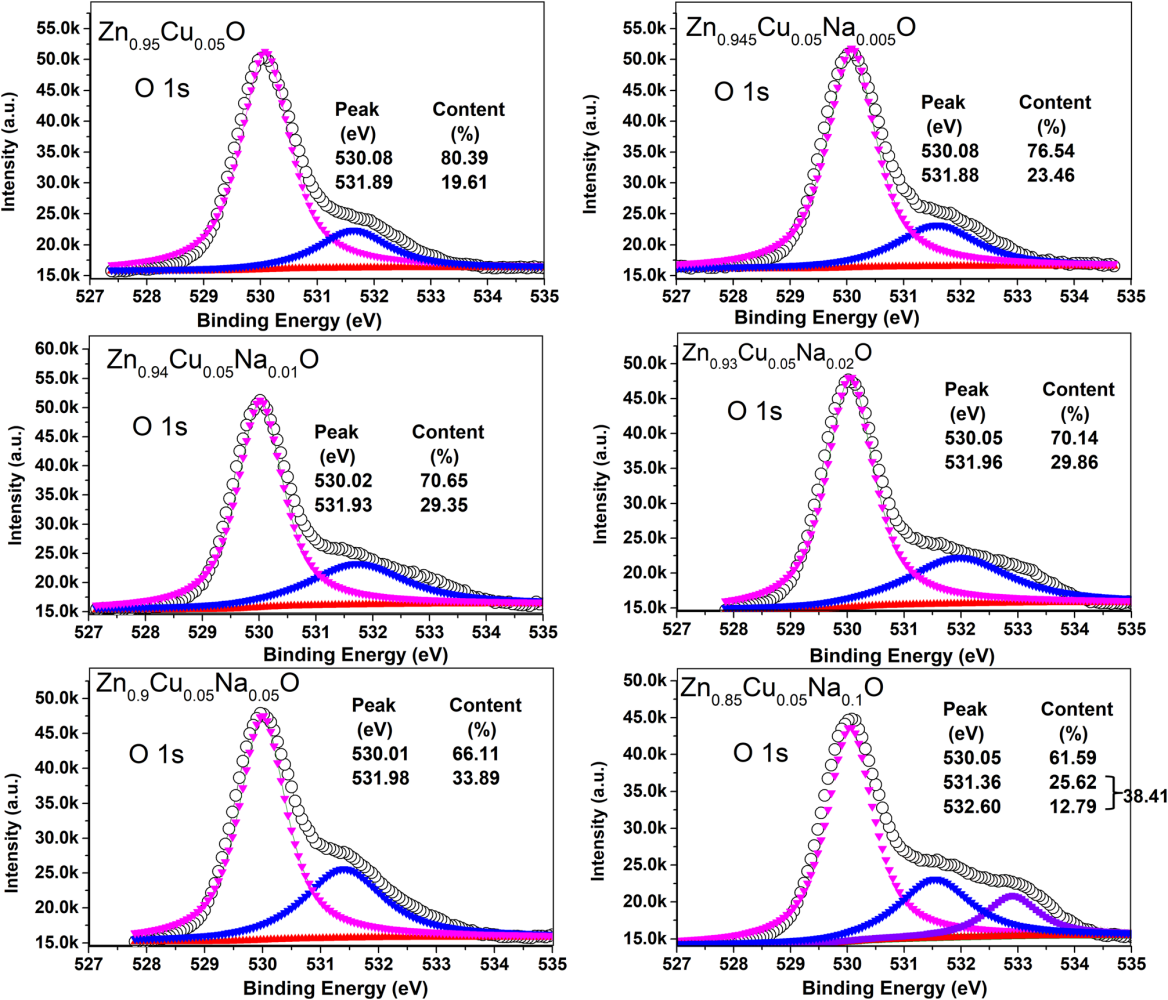
XPS of the O 1s core level and its corresponding Gaussian curve fittings for the Zn_0.95−*x*_Cu_0.05_Na_*x*_O nanocrystals.

**Fig. 6:**
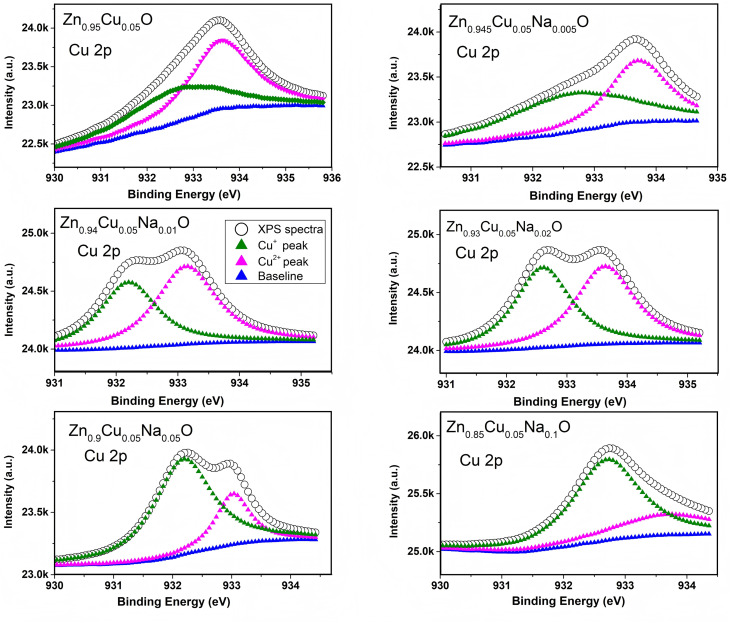
XPS of the Cu 2p core level and its corresponding Gaussian curve fittings for the Zn_0.95−*x*_Cu_0.05_Na_*x*_O nanocrystals.

The surface morphology of Zn_0.95−*x*_Cu_0.05_Na_*x*_O nanocrystals was characterized using scanning electron microscopy (SEM) images depicted in Fig. 2(a)–(f). The sample morphologies were not appreciably changed by doping with Na and Cu, which appeared quasi spherical. Moderate Na doping seemed to have improved crystal nanoparticle uniformity. In the HRTEM images of Zn_0.9_Cu_0.05_Na_0.05_O in Fig. 2(g) and (h), the nanomaterial exhibits large clusters of 100-500-nm particles due to the high treatment temperature, in which the interplanar lattice spacings of 0.235 and 0.283 nm belong to the (111) and (100) planes of CuO and ZnO, respectively. These findings are in agreement with the XPS results. XPS is a sensitive technique for assessing the electronic structure of the surfaces of solids.^31^ In the high-resolution Zn2p XPS spectra for Zn_0.95−*x*_Cu_0.05_Na_*x*_O nanocrystals with different Na concentrations, no significant variation in Zn chemical states from the typical Zn^2+^ state was observed for any sample (Fig. 3). Meanwhile, the Na 1s peaks of the 1072.1 eV binding energy of the XPS spectrum of Zn_0.95−*x*_Cu_0.05_Na_*x*_O did not change, regardless of whether the Na concentration was variable (Fig. 4). Remarkably, we observed that the O 1s XPS spectrum profile was quite asymmetric (Fig. 5). The spectrum could be resolved into two components centered at ∼530.1 eV and ∼531.9 eV using Gaussian functions. The low binding energy component of the O 1s spectrum is attributed to lattice oxygen in the ZnO matrix, while the higher binding energy peak corresponds to surface hydroxyl groups (OH–) associated with oxygen vacancies.^32^ It is worth noting that the high binding energy peak is in accordance with the Zn–O bond increasing in intensity with Na doping, suggesting that higher Na concentrations favor O^2−^ ion formation in the wurtzite structure of the hexagonal Zn^2+^ ion array. The Cu 2p_3/2_ spectra of the Cu^+^ peak was centered at 932.5 eV, whereas the Cu^2+^ peak of the binding energy was at 933.7 eV.^33,34^ In addition, the spectra demonstrated clearly that increasing Na concentration offsets Cu peaks slightly in the direction of lower binding energy (Fig. 6), suggesting that a high Na concentration favors the formation of samples with a high Cu^+^/Cu^2+^ ratio. Our results indicated that Cu valence changed significantly and Cu^+^ was in a dominant position when the Na doping concentration exceeded 10%. Combined with our previous study,^34^ we have found that the Cu^+^/Cu^2+^ ratio could be tuned by Na doping.

**Fig. 7:**
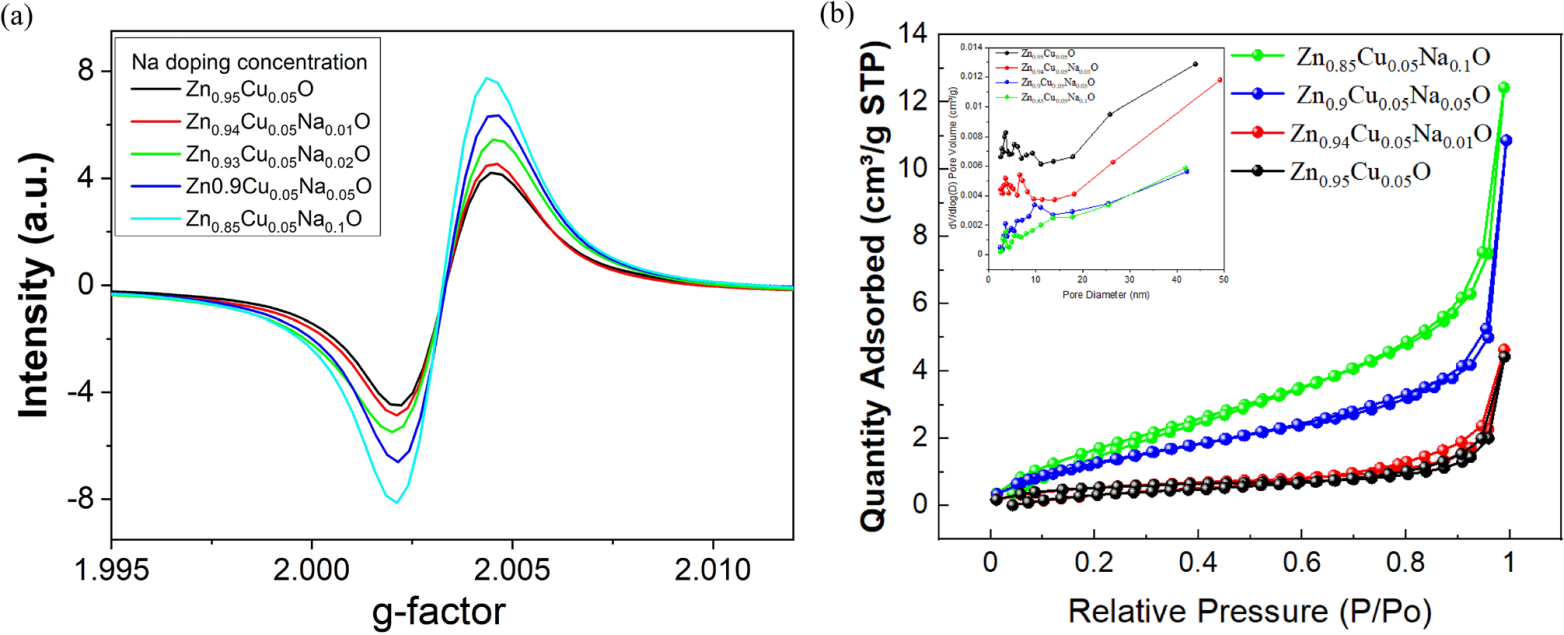
(a) Room-temperature EPR spectra of the Zn_0.95−*x*_Cu_0.05_Na_*x*_O-based sensors. (b) Isothermal curves of the Zn_0.95−*x*_Cu_0.05_Na_*x*_O nanocrystals (*x* = 0, 0.01, 0.02, 0.05, and 0.1) during the nitrogen adsorption–desorption process. The inset figure shows pore size distribution plot of the Zn_0.95−*x*_Cu_0.05_Na_*x*_O nanocrystals (*x* = 0, 0.01, 0.05, and 0.1).

EPR spectroscopy was employed to examine the paramagnetic characteristics of Zn_0.95−*x*_Cu_0.05_Na_*x*_O nanocrystals (Fig. 7(a)). The spectra revealed intrinsic defects of the gas-sensing nanocrystals. A strong EPR signal was observed centered at *g* = 2.0034 for Zn_0.95−*x*_Cu_0.05_Na_*x*_O, which could be ascribed to unpaired electrons trapped on oxygen vacancy sites. The stronger the EPR signal, the larger the concentration of oxygen vacancy defects. The EPR signal corresponds to the electrons trapped at surface O-vacancies, which facilitate the adsorption of oxygen molecules, reducing O_2_ to ˙O_2_. The signals of Zn_0.95−*x*_Cu_0.05_Na_*x*_O nanocrystals are stronger with increased Na doping, further confirming that the intrinsic defects of the samples increase with Na doping, in agreement with the XRD and XPS results. Considering these results together, we conclude that samples with a higher Cu^+^/Cu^2+^ ratio have more oxygen vacancy defects. A further understanding of the NO_2_ gas-sensing mechanism and the influence of Na^+^ on Zn_0.95−*x*_Cu_0.05_Na_*x*_O can be achieved through the acquisition of information on photogenerated charge carrier separation and carrier transport direction using the surface photovoltage technique. We have discussed in a previous study^35^ that ZnO is an n-type semiconductor with a positive SPV response due to photoinduced holes moving to the surface of ZnO. The stronger the SPV signal, the more photogenerated holes accumulate on the surface. That being said, the separation efficiency of photogenerated charges is higher with a higher SPV response, which will cause more O_2_^−^ to react with photo-generated charges. It is noteworthy that samples containing higher Na doping and lower Cu^2+^/Cu^+^ ratio allow for higher SPV intensity, suggesting that large amounts of Cu^+^ and oxygen vacancies enhance the separation of photo-generated charges of the photoelectric gas sensor.

Fig. 7(b) presents the nitrogen adsorption–desorption isotherms for Zn_0.95−*x*_Cu_0.05_Na_*x*_O nanocrystals. For all samples, the isotherms show a gentle increase in the low relative pressure region (*P*/*P*_o_ < 0.1), while a distinct H3-type hysteresis loop^36^ appears in the medium-to-high pressure range (*P*/*P*_o_ = 0.4–1.0), as seen in the inset of Fig. 7(b). The closure point of the hysteresis loop is located at *P*/*P*_o_ ≈ 0.42. Combined with the absence of a steep adsorption rise at low *P*/*P*_o_, this indicates that the materials lack microporous structures but contain open slit-shaped mesopores formed by the stacking of rod-like nanocrystals,^37^ which is consistent with the SEM observations in this study. Such stacked slit-like pore structures not only provide fast diffusion pathways for gas molecules but also offer abundant surface reaction sites, laying a structural foundation for excellent gas-sensing performance. The specific surface area was calculated using the Brunauer–Emmett–Teller (BET) theoretical model. The pore-size distribution was determined by analyzing the desorption branch data using the Barrett–Joyner–Halenda (BJH) model. The results indicate that Zn_0.95_Cu_0.05_O nanocrystals exhibit the highest specific surface area, approximately 5.4288 m^2^ g^−1^, while Zn_0.9_Cu_0.05_Na_0.05_O nanocrystals exhibit only about 1.7684 m^2^ g^−1^. Alev *et al.*^38^ reported that doping in ZnO can effectively regulate grain boundary migration and surface energy, thereby promoting the formation of nanostructures and the development of pore channels. In addition, Na^+^ may act as a flux during high-temperature synthesis, suppressing excessive grain growth and facilitating the preservation of more microporous structures. As evidenced by the data in Table 1, Zn_0.95_Cu_0.05_O possesses the highest specific surface area and the smallest average pore size. With increasing Na doping levels, the specific surface area of the samples initially decreased before exhibiting a slight recovery, while the average pore size showed a consistent upward trend. This behavior can be primarily ascribed to lattice distortion and altered grain growth dynamics induced by Na^+^ doping. Its incorporation leads to significant expansion of the ZnO lattice, generating numerous point defects and dislocations. Moreover, during high-temperature sintering, Na^+^ may act as a fluxing agent, lowering the activation energy for grain boundary migration and promoting the coalescence of smaller grains into larger ones. Consequently, the average particle size increases, accompanied by a reduction in specific surface area.

**Table 1:** Nitrogen adsorption–desorption isotherms for Zn_0.95−*x*_Cu_0.05_Na_*x*_O nanocrystals

Samples	Surface area/(m^2^ g^−1^)	Average pore sizes/nm	Pore volumes/(cm^3^ g^−1^)
Zn_0.95_Cu_0.05_O	5.4288 m^2^ g^−1^	12.2719 nm	0.017472 cm^3^ g^−1^
Zn_0.94_Cu_0.05_Na_0.01_O	3.7782 m^2^ g^−1^	16.1809 nm	0.015784 cm^3^ g^−1^
Zn_0.9_Cu_0.05_Na_0.05_O	1.7684 m^2^ g^−1^	16.9944 nm	0.006860 cm^3^ g^−1^
Zn_0.85_Cu_0.05_Na_0.1_O	2.1119 m^2^ g^−1^	20.5364 nm	0.006514 cm^3^ g^−1^

**Fig. 8:**
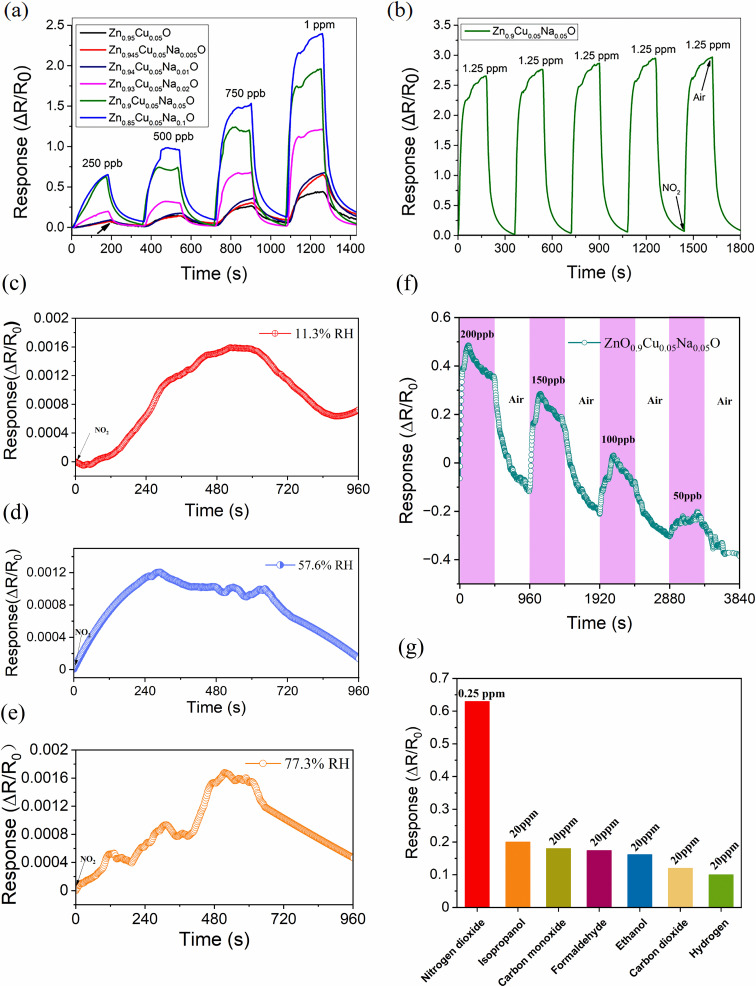
(a) Real-time sensing responses of the NO_2_ gas-sensing Zn_0.95−*x*_Cu_0.05_Na_*x*_O nanocrystals at room temperature (250 ppb, 500 ppb, 750 ppb and 1 ppm). (b) Repeatability of the Zn_0.9_Cu_0.05_Na_0.05_O sensor response to 1.25-ppm NO_2_ at room temperature. The sensing responses of the Zn_0.9_Cu_0.05_Na_0.05_O sensor to 2-ppm NO_2_ at (c) 11.3% relative humidity (RH), (d) 57.6% RH, and (e) 77.3% RH. (f) The sensing response curves of the Zn_0.9_Cu_0.05_Na_0.05_O sensors to NO_2_ at room temperature (50 ppb, 100 ppb, 150 ppb and 200 ppb). (g) Selectivity test of the Zn_0.9_Cu_0.05_Na_0.05_O gas sensor for different gases.

Combined with the gas-sensitive response test results in Fig. 8(a), although Zn_0_._95_Cu_0_._05_O exhibited the highest specific surface area, the Zn_0_._85_Cu_0_._05_Na_0_._1_O sample showed the best response to 1-ppm NO_2_ gas. This indicates that the gas-sensing performance depends not only on specific surface area but also on surface chemical activity and the distribution of surface oxygen species. Wang^39^ suggested that appropriately enlarging pore size facilitates rapid diffusion of gas molecules within the material, thereby reducing the response time. Therefore, the excellent gas-sensing performance of Zn_0_._85_Cu_0_._05_Na_0_._1_O could be attributed to its larger pore size (20.5364 nm), which promotes gas diffusion, along with Na doping that optimizes the surface electronic structure, synergistically improving the gas-sensing properties.

### 3.2 Gas-sensing performance and enhancement mechanism

The NO_2_ gas-sensing performances of Zn_0.95−*x*_Cu_0.05_Na_*x*_O-based sensors were investigated at room temperature. The comparative time dependence of the sensor response resistance shifts against 250 ppb, 500 ppb, 750 ppb, and 1 ppm NO_2_ under 365-nm light irradiation is shown in Fig. 8(a). The fabricated sensors demonstrated excellent sensing performance at room temperature with relatively stable baselines, as shown in Table 2. Table 3 shows the NO_2_ gas-sensing performance of different composite materials. The electrical resistance of the sensors enhances after exposure to NO_2_ and then recovers immediately once exposed to dry air. The dynamic response curves indicate that the sensors coated with a sensitive material containing a high Cu^+^/Cu^2+^ ratio due to higher Na doping concentration show higher sensitivity towards the tested gas compared with those coated with a material with a low Cu^+^/Cu^2+^ ratio, as shown in SPV. Even at 250 ppb NO_2_, the sensors coated with the sensitive material with a high Cu^+^/Cu^2+^ ratio showed stable response signals. Fig. 8(b) illustrates the repeatability of the Zn_0.9_Cu_0.05_Na_0.05_O nanoparticle-based sensor response for an NO_2_ gas concentration of 1.25 ppm at room temperature, where no obvious change in the amplitude of the sensing signal was seen over five cycles. Under relative humidity levels of 11.3%, 57.6%, and 77.3%, the Zn_0.9_Cu0_.05_Na_0.05_O sensor exhibited relative humidity-dependent responses to 2 ppm NO_2_, as shown in Fig. 8(c)–(e), respectively. The experimental results indicate that the fabricated Zn_0.9_Cu_0.05_Na_0.05_O sensor exhibits excellent sensing performance, achieving a NO_2_ detection limit of as low as 50 ppb, as shown in Fig. 8(f). In addition, the selectivity to NO_2_ was confirmed by testing gases, including isopropanol, carbon monoxide, formaldehyde, ethanol, carbon dioxide, and hydrogen (Fig. 8(f)). The sensor fabricated using Zn_0.9_Cu_0.05_Na_0.05_O exhibited a significantly stronger response to NO_2_ gas at 0.25 ppm compared with the other interference gases at 20 ppm. The corresponding sensitivity values were 0.63, 0.2, 0.18, 0.174, 0.162, 0.12, and 0.1, respectively. These results indicate that the Zn_0.9_Cu_0.05_Na_0.05_O sensor possesses favorable selectivity toward nitrogen dioxide.

**Table 2:** NO_2_ gas-sensing responses of each sample at varying concentrations

Response sample	250 ppb	500 ppb	750 ppb	1 ppm
Zn_0.95_Cu_0.05_O	8%	13%	25%	45%
Zn_0.945_Cu_0.05_Na_0.005_O	7%	13%	30%	66%
Zn_0.94_Cu_0.05_Na_0.01_O	10%	16%	37%	68%
Zn_0.93_Cu_0.05_Na_0.02_O	18%	34%	67%	125%
Zn_0.9_Cu_0.05_Na_0.05_O	63%	76%	125%	182%
Zn_0.85_Cu_0.05_Na_0.1_O	68%	100%	153%	241%

**Table 3:** Nitrogen dioxide gas-sensing performance of different composite materials

Sensor materials	Gas concentration/ppm	Operation temperature/°C	Response	References
Ni–ZnO	10	250	120.8%	40
ZnO–Au	10	200	40%	41
Li–ZnO	30	25	70.25%	42
Fe–ZnO	100	400	31.81%	43
rGO-CuO/ZnO	100	25	77%	44
Pd–ZnO	2	100	46.3%	45
CuO–ZnO	10	140	9.7%	46
Zr–ZnO	0.5	150	13.63%	47
Li–Cu: ZnO	75	210	2415.26%	48
Cu–ZnO	1	200	3.7%	49
Pd@Pt-ZnO	0.05	80	60.3%	50
Ce–ZnO	100	250	8.6%	51
CuO–ZnO	100	25	36.7%	52
Ni–ZnO	20	25	264%	53
Zn_0.9_Cu_0.05_Na_0.05_O	0.25	25	63%	This work

**Fig. 9:**
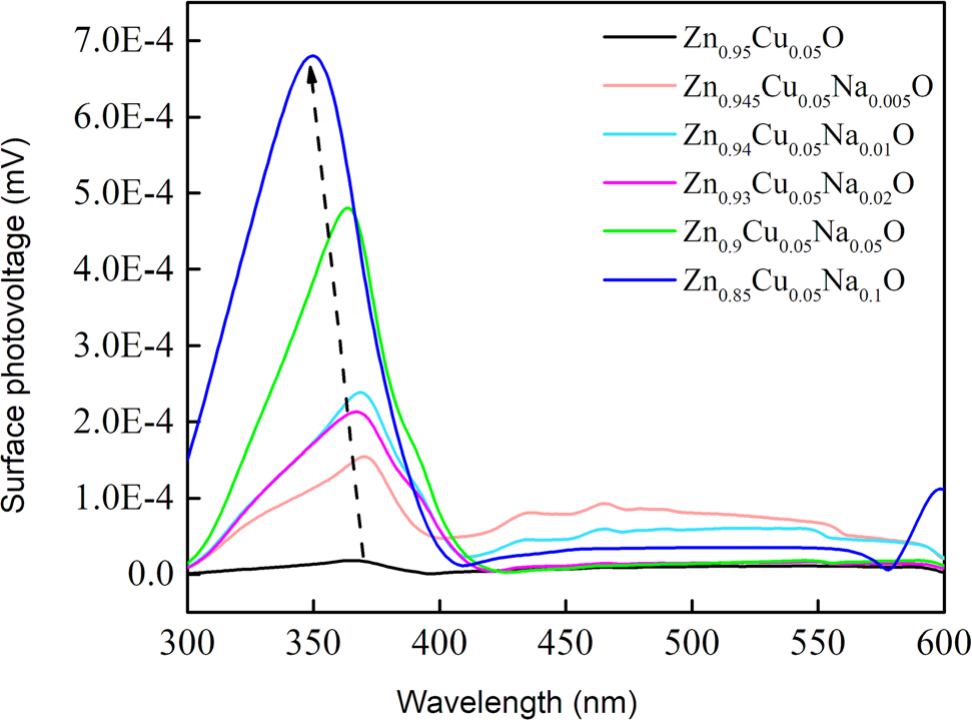
Surface photovoltage (SPV) spectra of the Zn_0.9_Cu_0.05_Na_0.05_O nanocrystals.

From the above results, we conclude that Na doping enhances the NO_2_ sensing performance of UV-activated sensors composed of Zn_0.95−*x*_Cu_0.05_Na_*x*_O nanocrystals. Further understanding of the role of Na ions in the NO_2_ sensing of light-activated Zn_0.95−*x*_Cu_0.05_Na_*x*_O nanocrystals requires an understanding of the separation of photo-generated charge carriers and their transfer direction. Such an information can be investigated *via* surface photovoltage analysis conducted in an air atmosphere. Fig. 9 presents the maximum photovoltage response of the Zn_0.95−*x*_Cu_0.05_Na_*x*_O nanocrystals, which occurs at 350–370 nm. It is noteworthy that the sample with a higher Cu^+^/Cu^2+^ ratio exhibits a stronger surface photovoltage intensity, suggesting that Na doping increases the separation of photo-generated charges. The rapid transfer of charge carriers is extremely important for photoelectric gas sensors. In air, the holes generated by photon absorption integrate with the nanoparticle surface oxygen ions, leading to the desorption of oxygen species. Furthermore, O_2_ captures the remaining electrons of separated electron–hole pairs to produce O_2_^−^. To reach equilibrium between the adsorption and desorption of O_2_, the electron depletion region beneath the sample surface changes at room temperature. Exposure to the stronger oxidizing NO_2_ widens the depletion layers by increasing resistance or decreasing electric current. When NO_2_ gas is removed, the electrons trapped in NO_2_^−^ and O_2_^−^ return to the surface of the nanocrystals to recombine with the holes, resulting in the narrowing of the depletion layer.

**Fig. 10:**
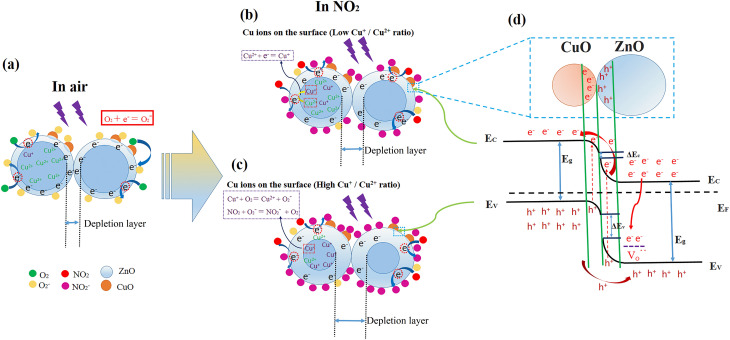
Schematic of the gas-sensing mechanism of Zn_0.95−*x*_Cu_0.05_Na_*x*_O nanocrystals responding to NO_2_ at room temperature.

In fact, extending the lifetime of electron–hole pairs is significant for light-assisted room temperature NO_2_ gas detection. As a consequence, the rapid recombination of photogenerated electrons and holes is inhibited by the synergistic effects of CuO decoration, oxygen vacancies, and Cu^+^/Cu^2+^ ratio in our experiments. ZnO/CuO heterojunction has been drawn according to the experimental data in Fig. 10. The light-assisted enhanced gas-sensing mechanism for our Zn_0.95−*x*_Cu_0.05_Na_*x*_O samples can be explained as follows: ZnO has a hexagonal wurtzite structure, which is a relatively open close-packed lattice. Na^+^ ions substitute for Zn in half of the tetrahedral sites, and more oxygen atoms are allowed to sit in the lattice sites, thereby generating oxygen vacancies. The substitution of Zn^2+^ with Na^+^ could introduce more defects and vacancies into the nanocrystal lattice. Surface oxygen vacancies can evoke a deeply trapped doubly charged oxygen vacancy (V_O_˙˙), which can capture the electrons beside the conduction band of ZnO to inhibit the recombination of photogenerated electron holes. Surface oxygen vacancies are correlated with Na doping, as demonstrated by the SPV results. NO_2_ adsorption of the samples, therefore, is increased with Na^+^ doping, thereby enhancing gas sensitivity. In ZnO, sodium (Na) substitutes for zinc (Zn) as a monovalent cation (Na^+^). The substitution of Na^+^ for the divalent Zn^2+^ introduces an effective negative charge, formally creating an acceptor defect denoted as Na_Zn. To maintain macroscopic charge neutrality, the system undergoes a charge compensation process, which in this case involves the reduction of a portion of the co-present copper ions from the Cu^2+^ to the Cu^+^ state. As the concentration of Na increases, the proportion of Cu^+^ ions rises concomitantly. These monovalent Cu^+^ ions subsequently occupy Zn^2+^ lattice sites, forming a defect center (Cu_Zn) that carries an effective single negative charge relative to the lattice. The accumulation of these negatively charged (Cu_Zn) centers necessitates further compensation to preserve the overall electrical neutrality of the crystal. A prevalent mechanism for this secondary compensation is the formation of intrinsic positively charged defects, specifically ionized oxygen vacancies (V_O_˙˙). The formation of these donor-like vacancies effectively balances the negative charge from the acceptor defects, thereby stabilizing the doped lattice.

On the other hand, Cu atoms are predicted to replace Zn within the ZnO lattice. The responses of the sensors are rapid. Zn_0.95−*x*_Cu_0.05_Na_*x*_O nanoparticle-based sensors with high Cu^+^/Cu^2+^ ratio demonstrate better gas-sensing responses compared with sensors with low Cu^+^/Cu^2+^ ratio. There is a direct correlation between gas concentration and electrical resistance change. Theoretically, the Zn_0.85_Cu_0.05_Na_0.1_O sensor can detect lower concentrations than the measured minimum of 250-ppb NO_2_. Cu^2+^ near the depletion layer and grain boundary can rapidly take up the photogenerated electrons to form Cu^+^ as follows:

1 Cu^2+^ + e^−^ = Cu^+^

This phenomenon leads to a wider depletion layer in Zn_0.95−*x*_Cu_0.05_Na_*x*_O nanoparticle-based sensors with a low Cu^+^/Cu^2+^ ratio (Fig. 10(b)). The variation of the Cu^+^/Cu^2+^ ratio upon electron attachment provides a continuous electron flux for the reduction reaction.^39,40^ Based on the XPS results, excessive Na^+^ induces the Cu valence state to change from +2 to +1 when the Na concentration exceeds 10%. These chemical changes can be described as follows:

2 Cu^+^ + O_2_ = Cu^+^ + O_2_^−^

3 NO_2_ + O_2_^−^ = NO_2_^−^+ O_2_

From reactions eqn (1)–(3), Cu^+^ and Cu^2+^ evidently play a pivotal role in the absorption and desorption of a tested gas on the sensing material's surface.

Based on the experimental data in this study, we can draw a schematic diagram of the ZnO/CuO heterojunction energy band (Fig. 10(c)). The work function of p-CuO is larger than that of n-ZnO with low electronic emission possibility,^41^ where the conduction band offset (Δ*E*_c_) of the p–n junction is about 0.12–0.3 eV. The generated free electrons in our samples could easily deliver the p–n junction due to the low Δ*E*_c_, and simultaneously, the holes in CuO will amass close to the VB of CuO due to the large value of the barrier height (Δ*E*_v_).^42^ We noticed that the electron mobility of CB will further improve because the Δ*E*_c_ value is lower than the Δ*E*_v_ value.^43,44^ Correspondingly, the higher the separation rate of the photoexcited electrons and holes, the more the electrons and holes participate in redox reactions. We find that the ZnO/CuO heterojunctions reveal in all samples, which should not be the main cause of gas sensitivity changes in this work. In view of the preceding discussion, it is now possible to ascertain how Cu ion valence state changes contribute to the modulation of the depletion layer to endow the devices with gas-sensing capabilities. For Zn_0.95−*x*_Cu_0.05_Na_*x*_O nanoparticle-based sensors with higher Cu^+^/Cu^2+^ ratios, the generated Cu^+^ leads to a higher density of surface-accumulated O^2−^. That is, the presence of large amounts of Cu^+^ is beneficial for the efficient modulation of the depletion layer. The Cu^+^/Cu^2+^ ratio affects the coverage degree of O_2_^−^, which directly influences NO_2_ gas responsivity.^54–59^

## 4. Conclusions

We herein propose a facile strategy to further enhance the gas-sensing performance of ZnO-based sensors. In this study, the efficient separation of electron–hole pairs is the main driving factor behind the enhancement of gas-sensing efficiency, resulting from the synergistic effects of high Cu^+^/Cu^2+^ ratio and surface oxygen vacancies. In particular, Zn_0.85_Cu_0.05_Na_0.1_O-based sensors show excellent gas-sensing response against NO_2_ at 250 ppb at room temperature. It is believed that the protocol can be devoted to designing other types of highly efficient ZnO-based sensors incorporating other metals cations with the valence shift, such as iron, manganese, cobalt, and vanadium.

## Conflicts of interest

There are no conflicts to declare.

## Data Availability

The data supporting this article have been included as part of the manuscript. For requests regarding the original data, please do not hesitate to contact the corresponding author.

## References

[cit1] Park J., Ghosh R., Song M. S., Hwang Y. J., Tchoe Y. B., Saroj R. K., Ali A., Guha P., Kim B., Kim S. W., Yi G. C. (2022). Individually addressable and flexible pressure sensor matrixes with ZnO nanotube arrays on graphene. NPG Asia Mater..

[cit2] Volkov V. V., Oliver D. J., Perry C. C. (2020). Polariton condensation and surface enhanced Raman in spherical ZnO microcrystals. Nat. Commun..

[cit3] Jiang X. H., Liu G., Tang L. P., Wang A. Z., Tian Y., Wang A., Du Z. L. (2020). Quantum dot light-emitting diodes with an Al-doped ZnO anode. Nanotechnology.

[cit4] Ouyang W. X., Chen J. X., Shi Z. F., Fang X. S. (2021). Self-powered UV photodetectors based on ZnO nanomaterials. Appl. Phys. Rev..

[cit5] Bang J., Kim Y. S., Park C. H., Gao F., Zhang S. B. (2014). Understanding the presence of vacancy clusters in ZnO from a kinetic perspective. Appl. Phys. Lett..

[cit6] Zheng M. J., Gui P. B., Wang X., Zhang G. Z., Wan J. X., Zhang H., Fang G. J., Wu H., Lin Q. Q., Liu C. (2019). ZnO ultraviolet photodetectors with an extremely high detectivity and short response time. Appl. Surf. Sci..

[cit7] Bhosale A. S., Abitkar K. K., Sadalage P. S., Pawar K. D., Garadkar K. M. (2021). Photocatalytic and antibacterial activities of ZnO nanoparticles synthesized by chemical method. J. Mater. Sci.:Mater. Electron..

[cit8] Jiang S. J., Lin K. L., Cai M. (2020). ZnO Nanomaterials: Current Advancements in Antibacterial Mechanisms and Applications. Front. Chem..

[cit9] Li Z. L., Li Z. Q., Zuo C. L., Fang X. S. (2022). Application of nanostructured TiO_2_ in UV photodetectors: a review. Adv. Mater..

[cit10] Banerjee D., Asuo I. M., Pignolet A., Nechache R., Cloutier S. G. (2020). High performance photodetectors using porous silicon-TiO_2_ heterostructure. Eng. Res. Express.

[cit11] Ge M. X., Xie D. Q., Jiao C., Yang Y. W., Shen L., Qiu M. B., Zhang H., He Z. J., Liang H. X., Tian Z. J. (2022). Mechanical properties and biocompatibility of MgO/Ca_3_(PO_4_)_2_ composite ceramic scaffold with high MgO content based on digital light processing. Ceram. Int..

[cit12] Gudkov S. V., Burmistrov D. E., Serov D. A., Rebezov M. B., Semenova A. A., Lisitsyn A. B. (2021). A Mini Review of Antibacterial Properties of ZnO Nanoparticles. Front. Phys..

[cit13] Sulaiman S., Izman S., Uday M. B., Omar M. F. (2022). Review on grain size effects on thermal conductivity in ZnO thermoelectric materials. RSC Adv..

[cit14] Giovannelli F., Chen C., Díaz-Chao P., Guilmeau E., Delorme F. (2018). Thermal conductivity and stability of Al-doped ZnO nanostructured ceramics. J. Eur. Ceram. Soc..

[cit15] Kaviyarasu K., Khamlich T., Magdalane C. M., Maazaab M. (2019). Stability and thermal conductivity of CuO nanowire for catalytic applications. J. Environ. Chem. Eng..

[cit16] Eisenmann T., Asenbauer J., Rezvani S. J., Diemant T., Behm R. J., Geiger D., Kaiser U., Passerini S., Bresser D. (2021). Impact of the transition metal dopant in zinc oxide lithium-ion anodes on the solid electrolyte interphase formation. Small Methods.

[cit17] Soltani S., Akhbari K., White J. (2020). Synthesis, crystal structure, magnetic, photoluminescence and antibacterial properties of dinuclear Copper (II) complex. J. Mol. Struct..

[cit18] Roguai S., Djelloul A. (2020). A structural and optical properties of Cu-doped ZnO films prepared by spray pyrolysis. Appl. Phys. A.

[cit19] Ma Q., Yang X., Lv X., Jia H., Wang Y. (2019). Cu doped ZnO hierarchical nanostructures: morphological evolution and photocatalytic property. J. Mater. Sci.: Mater. Electron..

[cit20] Brahma S., Yeh Y. W., Huang J. L., Liu C. P. (2021). Cu-doped p-type ZnO nanostructures as unique acetone sensor at room temperature (∼25 °C). Appl. Surf. Sci..

[cit21] Ganesh R. S., Durgadevi E., Navaneethan M., Patil V. L., Ponnusamy S., Muthamizhchelvan C., Kawasaki S., Patil P. S., Hayakawa Y. (2018). Tuning the selectivity of NH_3_ gas sensing response using Cu-doped ZnO nanostructures. Sens. Actuators, A.

[cit22] Wu C., Huang Q. (2010). Synthesis of Na-doped ZnO nanowires and their photocatalytic properties. J. Lumin..

[cit23] Sáaedi A., Yousefi R. (2017). Improvement of gas-sensing performance of ZnO nanorods by group-I elements doping. J. Appl. Phys..

[cit24] Elsayed M. H., Elmorsi T. M., Abuelela A. M., Hassan A. E., Alhakemy A. Z., Bakr M. F., Chou H. H. (2020). Direct sunlight-active Na-doped ZnO photocatalyst for the mineralization of organic pollutants at different pH mediums. J. Taiwan Inst. Chem. Eng..

[cit25] Jaisutti R., Lee M. Y., Kim J., Choi S., HA T. J., Kim J., Kim H., Park S. K., Kim Y. H. (2017). Ultrasensitive room-temperature operable gas sensors using p-type Na:ZnO nanoflowers for diabetes detection. ACS Appl. Mater. Interfaces.

[cit26] Yuan H., Xu M., Dong C. J., Ma J., Wang X. Y. (2019). Mechanistic insights into magnetic and gas sensing properties of (F, Na)-codoped ZnO nanocrystals by room-temperature. Appl. Surf. Sci..

[cit27] Zhang Q. P., Pang Z. M., Hu W. Y., Li J., Liu Y. T., Yu F., Zhang C. W., Xu M. (2021). Performance degradation mechanism of the light-activated room temperature NO_2_ gas sensor based on Ag-ZnO nanoparticles. Appl. Surf. Sci..

[cit28] Zhang Q. P., Xie G. Z., Xu M., Su Y. J., Tai H. L., Du H. F., Jiang Y. D. (2017). Visible light-assisted room temperature gas sensing with ZnO-Ag heterostructure nanoparticles. Sens. Actuators, B.

[cit29] Xu M., Yuan H., You B., Zhou P. F., Dong C. J., Duan M. Y. (2014). Structural, optical, and magnetic properties of (Co, Cu)-codoped ZnO films with different Co concentrations. J. Appl. Phys..

[cit30] Shen J. L., Jiang S., Xu Y. C., Li M. Y., Zhu S., Chen Z. B., Lin X. F., Liu H., Li H. L., Zhang J. Z. (2017). Boron and sodium co-doped ZnO varistor with high stability of pulse current surge. J. Alloys Compd..

[cit31] Ganapathi K., Kaur M., Pathak M., Shaheera M., Pathak A., Gadkari S. C., Debnath A. K. (2021). Highly sensitive NO_2_ sensor based on ZnO nanostructured thin film prepared by SILAR technique. Sens. Actuators, B.

[cit32] Han Z., Ren L., Cui Z., Chen C., Pan H., Chen J. (2012). Ag/ZnO flower heterostructures as a visible-light driven photocatalyst via surface plasmon resonance. Appl. Catal., B.

[cit33] Vij A., Gautam S., Won S. O., Thakur A., Lee I. J., Chae K. H. (2012). X-ray photoelectron spectroscopy of Zn0.98Cu0.02O thin film grown on ZnO seed layer by RF sputtering. Mater. Lett..

[cit34] Yuan H., Xu M., Du X. S. (2015). Effects of Co doping on the structural and optical properties of ZnCuO thin films. Mater. Lett..

[cit35] Wang J. Y., Deng J. H., Li Y. B., Yuan H., Xu M. (2020). ZnO nanocrystal-coated MoS_2_ nanosheets with enhanced ultraviolet light gas sensitive activity studied by surface photovoltage technique. Ceram. Int..

[cit36] Thommes M., Kaneko K., Neimark A. V., Olivier J. P., Rodriguez-Reinoso F., Rouquerol J., Sing K. S. W. (2015). Physisorption of gases, with special reference to the evaluation of surface area and pore size distribution. Pure Appl. Chem..

[cit37] Zhang J., Wang S. R., Xu M. J., Wang Y., Zhu B. L., Zhang S. M., Huang W. P., Wu S. H. (2013). Hierarchical porous ZnO architectures for gas sensing application. CrystEngComm.

[cit38] Alev O., Ergün İ., Özdemir O., Çolakerol Arslan L., Büyükköse S., Öztürk Z. Z. (2021). Enhanced ethanol sensing performance of Cu-doped ZnO nanorods. Mater. Sci. Semicond. Process..

[cit39] Wang J., Hu C. Y., Xia Y., Zhang B. (2020). Mesoporous ZnO nanostructures with highly exposed (0001) facets for ultra-sensitive gas sensors. J. Mater. Chem. A.

[cit40] Oum W., Mirzaei A., Shin Y. K. (2025). *et al.*, Enhancement of NO_2_ sensing of ZnO by irradiating with high-energy Ni ions. Sens. Actuators, B.

[cit41] Pathak A., Samanta S., Bahadur J. (2025). *et al.*, Tailoring ZnO-Au nano-composites for enhanced NO_2_ sensing: Synergistic effect of facet orientation and Au nano-particle surface functionalization. Appl. Surf. Sci..

[cit42] Singh N., Singh N., Bhargav A. (2024). *et al.*, Li-substituted ZnO nanoparticles exhibiting room temperature optical gas sensing for NO_2_ with swift response and recovery. Opt. Quant. Electron..

[cit43] Hiremath V. M., Momin N., Kangralkar V. M. (2024). *et al.*, Synthesis and characterization of Fe-doped ZnO films for enhanced NO_2_ gas-sensing applications. J. Korean Phys. Soc..

[cit44] Yadav D., Shukla S. P., Varma D. G. (2024). Synthesis of rGO-CuO/ZnO nanocomposites for humidity tolerant room temperature NO_2_ gas sensor. Appl. Phys. A.

[cit45] Luu M. H., Pham T. T. T., Nguyen D. V. (2024). *et al.*, Excellent NO_2_ sensor based on porous Pd-ZnO nanorods prepared by a facile hydrothermal method. Adv. Nat. Sci. Nanosci. Nanotechnol..

[cit46] Li S., Yu L., Zhang C. (2024). *et al.*, Controllable synthesis of heterostructured CuO–ZnO microspheres for NO_2_ gas sensors. Sens. Actuators, B.

[cit47] Rodrigues J., Tripathy S., Shimpi G. N. (2024). Enhanced performance of 1D rod shaped Zirconium (Zr) decorated over spherical shaped Zinc oxide (ZnO) nanostructures for NO_2_ gas. Opt. Mater..

[cit48] Jasmi K. K., Johny A. T., Siril S. V. (2024). *et al.*, Influence of lithium on Cu-doped ZnO thin films fabricated via sol-gel spin coating technique for improved NO_2_ gas sensing applications. J. Electroceram..

[cit49] Umar A., Ibrahim A. A., Begi N. A. (2024). *et al.*, Synthesis of Bitter gourd-shaped Cu-doped ZnO
nanostructures and their investigation for the detection of NO_2_ gas at low concentrations. Ceram. Int..

[cit50] Sui C., Zhang M., Li Y. (2024). *et al.*, Pd@Pt Core-Shell Nanocrystal-Decorated ZnO Nanosheets for ppt-Level NO_2_ Detection. ACS Sens..

[cit51] Ahmad U., Sheikh A., Rajesh K. (2023). *et al.*, Ce-doped ZnO nanostructures: A promising platform for NO_2_ gas sensing. Chemosphere.

[cit52] Smriti S., Rita D., Suman R. (2023). *et al.*, The development of CuO-ZnO based heterojunction for detection of NO_2_ gas at room temperature. Appl. Phys. A.

[cit53] Feng Y. H., Hong W., Di L. (2023). *et al.*, Pompon-like Ni doped ZnO mesoporous microspheres for conductometric NO_2_ sensing properties at room temperature. Sens. Actuators, B.

[cit54] Chen X. Y., Kuo D. H. (2017). Nanoflower bimetal CuInOS oxysulfide catalyst for the reduction of Cr (VI) in the dark. ACS Sustain. Chem. Eng..

[cit55] Riha S. C., Johnson D. C., Prieto A. Y. (2011). Cu_2_Se nanoparticles with tunable electronic properties due to a controlled solid-state phase transition driven by copper oxidation and cationic conduction. J. Am. Chem. Soc..

[cit56] Park S., Ko H., Kim S., Lee C. (2014). Role of the Interfaces in multiple networked one-dimensional core-shell nanostructured gas sensors. ACS Appl. Mater. Interfaces.

[cit57] Wang C., Fu X. Q., Xue X. Y., Wang Y. G., Wang T. H. (2007). Surface accumulation conduction controlled sensing characteristic of p-type CuO nanorods induced by oxygen adsorption. Nanotechnology.

[cit58] Thirumoorthi M., ShekDhavu S., Ganesh V., Abdulaal T. H., Yahia I. S., Deivatamil D. (2022). High responsivity n-ZnO/p-CuO heterojunction thin film synthesised by low-cost SILAR method for photodiode applications. Opt. Mater..

[cit59] Subha P. P., Jayaraj M. K. (2019). Enhanced room temperature gas sensing properties of low temperature solution processed ZnO/CuO heterojunction. BMC Chem..

